# Aβ levels in the jugular vein and high molecular weight Aβ oligomer levels in CSF can be used as biomarkers to indicate the anti-amyloid effect of IVIg for Alzheimer’s disease

**DOI:** 10.1371/journal.pone.0174630

**Published:** 2017-04-10

**Authors:** Takashi Kasai, Masaki Kondo, Ryotaro Ishii, Akihiro Tanaka, Suzuka Ataka, Hiroyuki Shimada, Takami Tomiyama, Hiroshi Mori, Mark Taylor, David Allsop, Masanori Nakagawa, Toshiki Mizuno, Takahiko Tokuda

**Affiliations:** 1 Department of Neurology, Research Institute for Geriatrics, Kyoto Prefectural University of Medicine,Kyoto, Japan; 2 Department of Geriatrics and Neurology, Osaka City University graduate school of medicine, Osaka, Japan; 3 Department of Neuroscience, Osaka City University graduate school of medicine, Osaka, Japan; 4 Division of Biomedical and Life Sciences, Faculty of Health and Medicine, Lancaster University, Lancaster, United Kindom; 5 North Medical Center, Kyoto Prefectural University of Medicine, Kyoto, Japan; 6 Department of Molecular Pathobiology of Brain Diseases, Research Institute for Geriatrics, Kyoto Prefectural University of Medicine, Kyoto, Japan; University of California San Diego, UNITED STATES

## Abstract

Intravenous immunoglobulin (IVIg) has been a candidate as a potential anti-amyloid immunotherapy for Alzheimer disease (AD) because it contains anti-amyloid β (Aβ) antibodies. Although several studies with IVIg in AD have been published, changing levels of Aβ efflux from the brain, or disaggregation of Aβ species induced by immunotherapy, have not been properly investigated. Here, we carried out an open label study of therapy with IVIg in five patients with AD. We collected plasma from a peripheral vein (peripheral-plasma) and from the internal jugular vein (jugular-plasma) to estimate directly the efflux of soluble Aβ from the brain. We also measured high molecular weight (HMW) Aβ oligomers in CSF as a marker to detect disaggregated Aβ. IVIg infusions were well tolerated in the majority of cases. However, one study subject had epileptic seizures after IVIg. Levels of HMW CSF Aβ oligomers in all participants were significantly increased after IVIg. Aβ_40_ and Aβ_42_ levels in jugular-plasma were continuously or temporarily elevated after treatment in three of five patients who showed preserved cognitive function, whereas levels of those in peripheral-plasma did not correlate with reactivity to the treatment. Other conventional biomarkers including ^11^C-Pittsburgh compound B retention were not altered after the treatment. These findings imply that HMW Aβ oligomer levels could be a better biomarker to reflect the anti-amyloid effects of IVIg than conventional Aβ species; moreover, Aβ in jugular-plasma seems to be a more direct and precise biomarker to estimate clearance of Aβ from the brain rather than Aβ in peripheral-plasma.

**Trial registration:** UMIN000022319

## Introduction

Alzheimer’s disease (AD) is the most common cause of dementia in elderly people but the available symptomatic drug treatments for this disease do not have any long-term effect [1]. Over the last decade, passive immunization using anti-amyloid β (Aβ) antibodies has held great promise as a potential new disease modifying therapy for AD. The principle of passive immunotherapy in AD is to reduce the levels of toxic Aβ species in the brain. Three molecular mechanisms for immunotherapy in AD have been generally postulated: increased efflux of Aβ from the brain by a ‘peripheral sink’ mechanism etc. [2]; the disaggregation of fibrillar and/or oligomeric Aβ in the brain [3]; and inhibition of Aβ aggregation[4]. Several studies suggest that passive immunization reverses cognitive deficits and reduces the load of cerebral Aβ in transgenic mouse models of AD [2, 5, 6] but no phase 3 trial of passive immunotherapy with positive results has been reported in human AD [7, 8]. This difference in response to immunotherapy between transgenic mice and humans could be caused by cerebrovascular aging, including atherosclerosis, which is seldom observed in mice, even in aged transgenic mouse models. Such cerebrovascular dysfunction could disturb the efflux of soluble Aβ from the brain and hinder the effects of immunotherapy. The other reason for the failure of clinical trials may be lack of good surrogate biomarkers measuring the anti-amyloid effects of drugs. It has been scarcely investigated whether anti-Aβ antibodies are sufficient to dissolve or to remove amyloid from the humans. If such a biomarker were available in the trial to exclude poor responders, the trial might have shown some success. Consequently, we propose that the following two considerations are important in pursuing clinical trials of passive immunization for AD. The first is that relatively young patients with AD should be included in these studies to avoid any possible interference due to cerebrovascular disease. The second is that the performance of the solubilization, mobilization and clearance of aggregated Aβ species induced by immunotherapy should be directly and precisely measured.

Intravenous immunoglobulin (IVIg) is a fractionated blood product that has been used to treat several medical conditions [9]and contains naturally occurring antibodies directed against Aβ [10, 11]. Therefore, IVIg has been a promising candidate as an anti-amyloid passive immunotherapy for AD, having high safety [12–14]. Although this treatment did not significantly slow the rate of cognitive decline in patients with AD in recent phase 3 trials, treatment with IVIg earlier in the course of the disease could still be beneficial [15]. Here, we conducted an open-label study of add on therapy with IVIg in five patients with AD. As mentioned above, we enrolled relatively young AD patients to avoid interference by atherosclerosis on Aß clearance. Then, we drew blood samples not only from a peripheral vein but also from the internal jugular vein to directly estimate brain Aβ clearance by IVIg. Moreover, we also collected cerebrospinal fluid (CSF) to measure disaggregated Aß species. We measured high molecular weight (HMW) Aβ oligomers in CSF as a more suitable biomarker to detect disaggregated Aβ species than conventional markers of Aβ[16]. The primary objectives of this study are, firstly, to compare the levels of Aβ (Aβ_40_ and Aβ_42_) in plasma from a peripheral vein (peripheral-plasma) and from the internal jugular vein (jugular-plasma) during the treatment; secondly, to assess the change in fibrillar Aβ load in patients with AD treated with IVIg: and thirdly to examine changes in levels of HMW Aβ oligomers in CSF during the treatment. The outcomes are investigating changes of conventional biomarkers for AD: Aβ_42_, total tau (t-tau), and 181-phosphorylated tau (p-tau) in CSF; amyloid deposition as measured by ^11^C-Pittsburgh compound B positron emission tomography (PIB-PET): and HMW Aβ oligomers as measured with our originally developed ELISA [16]. Neuropsychological tests, including mini-mental state examination (MMSE), were administered throughout the observational period to identify any changes in cognitive function.

## Material and methods

### Study design, ethics statement, and subject recruitment

**Fig 1:**
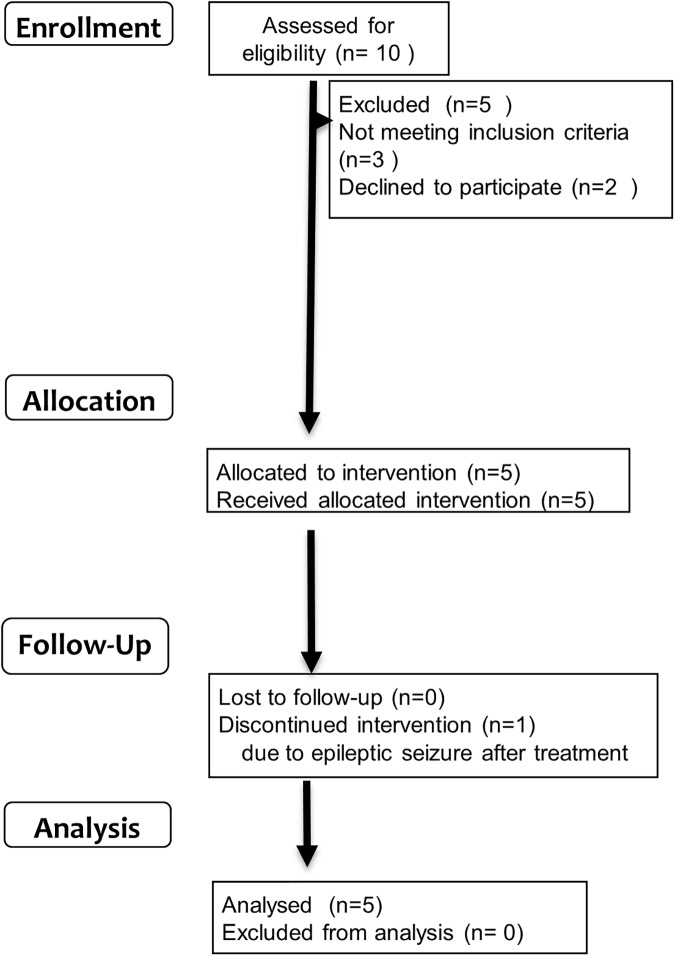
Trial schedule (A).

**Fig 2:**
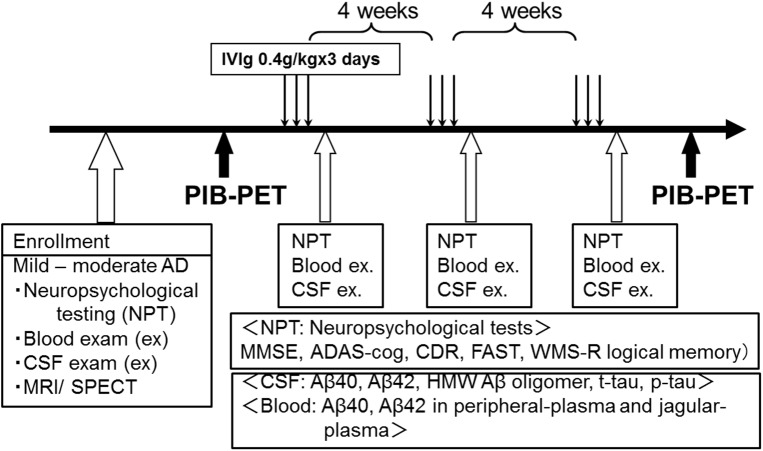
Flow diagram of the trial (B).

Our study was designed as an open label trial of IVIg in patients with mild and moderate AD that included 2 months of active treatment followed by at least two years of observation with neuropsychological tests. The study complied with the Declaration of Helsinki and was approved by the University Ethics Committee on 15^th^ Aug in 2008 (Kyoto Prefectural University of Medicine, 602–8566, Kyoto, Japan). The trial was conducted in accordance with Good Clinical Research practice. This study has been retrospectively registered at UMIN (University hospital Medical Information Network) Clinical Trials Registry on 16^th^ May 2016, because trial registration was not common in Japan when the trial was planned. The registration identification number is UMIN000022319 (http://www.umin.ac.jp/ctr/index-j.htm). Participants were recruited from Jul 2008 to Jan 2011. Written informed consent was obtained from the participants as well as caregivers for publication of their individual details and accompanying images in this manuscript. Five patients with AD were enrolled, based on the criteria described in the following subsection. All of the participants were given three cycles of IVIg in eight weeks (0.4 g/kg daily of Venoglobulin IH (Japan Blood Products Organization, Tokyo, Japan) for 3 days every four weeks). IVIg infusions were administrated through a 22 gauge intravenous catheter placed in a vein of the left arm. IVIg was mixed and prepared up to 2h prior to administration. Certified infusion pumps were employed to regulate rates of IVIg delivery. Total infusion times were 4–6 h per session depending on the patient’s body mass (Fig 1). Infusion process was performed under careful observation by certificated neurologists (TK, MK, RI, AT, or TT). Enrollment of patients, clinical assessment, treatment with IVIg, and specimen collection were performed at Kyoto Prefectural University of Medicine. PIB-PET was performed at Osaka City University graduate school of medicine. Participants were followed for at least 2 years (Fig 2). No financial reward was offered to the participants. (Research implementation plan and TREND statement check list of this study are in S1–S3 Files)

### Clinical assessments

All subjects underwent neurological examinations including cognitive testing and examinations of blood (from peripheral and internal jugular veins) and CSF at baseline and at every IVIg treatment thereafter. Brain magnetic resonance imaging (MRI) (a 1.5 Tesla MRI unit, Philips Intera Achieva Pulser scanner) was carried out at baseline.

PIB-PET scans were performed at one month before the first treatment and within four weeks after the final treatment. Inclusion criteria included a diagnosis of probable AD by the National Institute of Neurological and Communicative Disorders and Stroke (NINCDS-ARDRA) criteria, Aβ deposition in PIB-PET, stable doses of approved AD treatment for at least 3 months, ability to comply with the study protocol, and availability of a suitable caregiver. Subjects were excluded if they had vascular dementia as detected by MRI, known allergy to IVIg therapy, IgA deficiency, renal failure, untreated vascular risk factors, unstable co-morbid medical conditions, or were receiving other investigational AD therapies or certain psychoactive medications. Subjects were also screened to exclude the presence of significant depression, psychosis and other neuropsychiatric disorders. Serum viscosity, IgA levels, renal function, thyroid function, complete blood count, metabolic profile, and hepatitis virus titers were checked at baseline. Subsets of these tests were repeated throughout the study for safety purposes. MMSE, Alzheimer's Disease Assessment Scale-cognitive subscale (ADAS-cog), Clinical Dementia Rating (CDR), Functional Assessment Staging (FAST) and Wechsler Memory Scale Revised (WMS-R) logical memory testing was performed by the same rater at baseline and just before each cycle of IVIg. Thereafter, MMSE was carried out every 3 or 6 months throughout the following observational period (at least two years). Adverse events in accordance with the definition of the International Council for Harmonisation of Technical Requirements for Pharmaceuticals for Human Use (ICH) guideline were recorded.

### Sample collection

Sample collection was performed at a fixed time in the morning to avoid any effect from fluctuation of Aβ levels during the day [17]. Blood samples were drawn with 22 gauge needles simultaneously from the right internal jugular vein under ultrasound guidance and from the right median cubital vein through venous puncture, and then immediately transferred into the EDTA-containing tubes used to collect plasma samples. CSF was obtained through lumbar puncture at the L3/L4 or L4/L5 interspace, according to a standard protocol. Tubes were centrifuged at 1000 x g, plasma and CSF separated, aliquoted into 2 ml of polypropylene vials, and stored at– 80°C until time of analysis. Blood and CSF sampling were done at baseline, just before each cycle of IVIg, and at post IVIg (at least one month after the final cycle of treatment).

### Biomarker analyses

Aβ_42_, t-tau and p-tau concentration in CSF were quantified by Luminex assays with INNO-BIA AlzBio3 (INNOGENETICS Inc, Ghent, Belgium), as described by the manufacturer. Levels of HWM Aβ oligomers in CSF were measured with the BAN50- Single Antibody Sandwich–ELISA (BAN50 SAS-ELISA) as described previously [16]. This ELISA system employs the same monoclonal antibody (BAN50) for both capture and detection of the oligomers and cannot detect Aß monomers because the capture antibody would then occupy the only antibody-binding site available. This system does, however, detect Aß oligomers because they have multiple binding sites. This ELISA is specific only for HMW Aß oligomers of 10-20mer in size. Neither low molecular weight Aß oligomers nor Aß monomer is not detected [16]. Levels of HMW Aβ oligomers are shown as arbitrary units based on the concentration of the synthetic ‘multiple-antigenic’ peptide used for calibration of this ELISA. The signal per one arbitrary unit of this standard has been estimated to correspond to 1.54–5.0 pM of HMW Aß_42_ oligomers [18]. Aβ_40_ and Aβ_42_ in plasma were measured with INNO-BIA plasma Aß kits (INNOGENETICS Inc, Ghent, Belgium).

### PET scan

^11^C-PIB was synthesized using 2-(4’-aminophenyl)-6-hydroxybenzothiazole as the labeled precursor molecule. After an intravenous injection of ^11^C-PIB (150–300 MBq), a dynamic 60 min list-mode emission scan was acquired in the three-dimensional mode without arterial sampling using an Eminence-B PET scanner (Shimadzu Corp., Kyoto, Japan). According to the study performed by Lopresti et. al. [19], in which the frame summation of the dynamic images was recorded for 40–60 min., Logan graphical analysis was used for determining the regional counts (distribution volume ratio, DVR = binding potential + 1) using the cerebellum as the reference region. For this purpose, the cortical lesions occurring in the frontal, parietal, and lateral temporal lobes, and the precuneus regions were selected. The mean cortical DVR (MCDVR) was the mean of the DVR values of these lesions [20].

### Statistics

Differences between biomarker levels at baseline and post treatment were tested by a Wilcoxon signed-rank test using SPSS software version 23. Statistical significance was defined as p < 0.05. Statistical power for the test (1-β) was calculated by postulating their effect sizes were medium (0.5) using G*Power 3 software.

## Results

### Demographics

Five patients (3 male, 2 female) with probable AD participated in this study. At baseline, their ages ranged from 54 to 67 years (mean 60.8 years). MMSE scores ranged from 8 to 25 (mean 18.6). ADAS-cog scale ranged from 3.6 to 33.3 (mean 15.7). All subjects were receiving stable doses of a cholinesterase inhibitor (donepezil) for at least 3 months prior to enrolling in the study (Table 1). The median length of follow-up was 34 months (30–50).

**Table 1:** Baseline characteristics of patients.

Patient No.	Sex	Age	Duration of symptoms (years)	Use of choline esterase inhibitor	MMSE	ADAS-cog
1	M	63	3	Donepezil 5mg/day 24 months	8	33.3
2	F	63	1	Donepezil 3mg/day 18 months	17	11.7
3	F	67	1	Donepezil 5mg/day 3 months	23	18.3
4	M	57	3	Donepezil 5mg/day 11 months	20	11.7
5	M	54	1	Donepezil 5mg/day 12 months	25	3.6
	Mean±SD	60.8±5.2			18.6±6.7	15.7±11.1

SD: standard deviation

### Adverse events

IVIg infusions were well tolerated in the majority of cases. However, case 5 was admitted with a first attack of epileptic seizure seven days after the first cycle of IVIg. Neurological examination revealed no focal signs. Routine blood and CSF examinations were all within normal ranges, and brain MRI did not show any signs of encephalitis or amyloid-related imaging abnormalities (ARIA). Electroencephalogram on the 3rd hospital day showed no epileptic discharges. He did not have any history of previous epilepsy. At that time, there was a possibility that his epileptic seizure might be related to the IVIg treatment, and further administration of IVIg was accordingly discontinued. Although we discontinued IVIg, he developed a second generalized tonic-clonic seizure 3 months later. We therefore consider that his seizure is not related to IVIg therapy, but is caused by AD itself [21]. Other minor events including headaches (in case 3) that were judged to be treatment-related were benign and resolved spontaneously.

### MMSE

**Fig 3:**
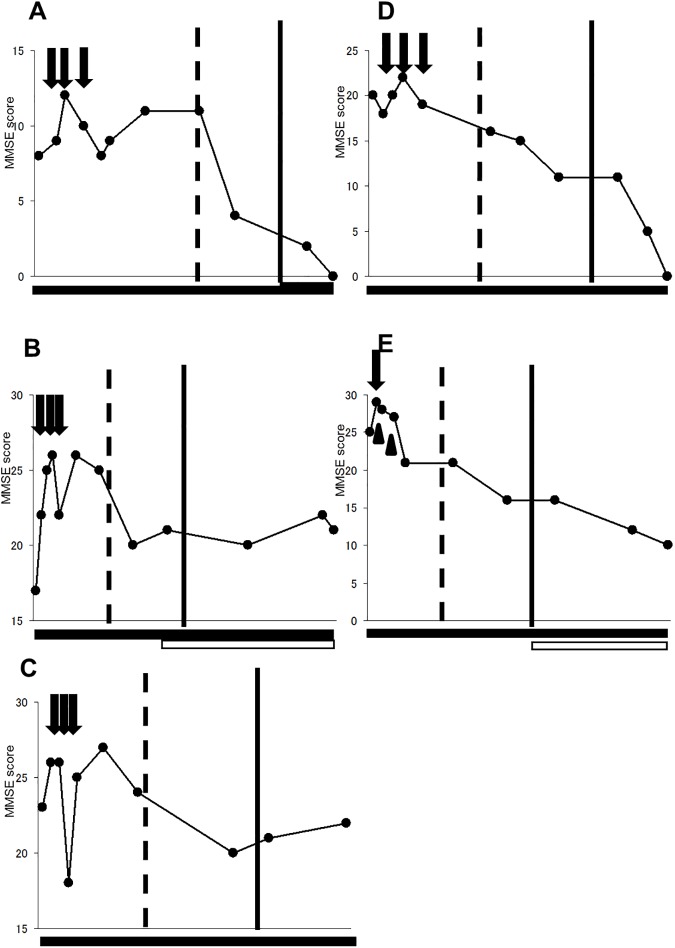
The MMSE scores over the observational period. A, B, C, D, and E show the MMSE changes in cases 1, 2, 3, 4, and 5, respectively. Arrows indicate IVIg treatments. Arrow heads indicate seizure attacks in case 5. Dashed lines and solid lines indicate 1 and 2 years after the first cycle of IVIg treatment in each patient. Bars below the graphs show the use of currently available symptomatic drugs for AD. Black bar: donepezil (thickness of bars indicates dosage of donepezil; thin bar = 3mg per day, medium bar = 5mg per day, bold bar = 8mg per day). White bar: memantine.

MMSE scores in patients during the period of treatment and the observational period are shown in the Fig 3. Changes of other cognitive scaling (ADAS-cog, CDR, FAST and WMS-R logical memory II) are summarized in Table A in S4 File. All patients including case 5 who had epilepsy and received incomplete treatment showed temporary improvement in MMSE scores during and/or after the three cycles of IVIg compared with their scores at the baseline. During one year after the treatment, during which the symptomatic drug treatments for AD in each patient were not changed, three (case 1. 2. 3) of four patients who received three cycles of IVIg therapy maintained improved MMSE scores compared with those at the baseline. These three patients are referred to as ‘good responders’ to IVIg in the following description. Case 4 who showed continuous deterioration of MMSE score after the treatment is referred as a ‘poor responder’ to IVIg. We could not judge the response to IVIg in case 5 due to the suspension of treatment, and in this case the MMSE score constantly deteriorated during the observational period.

### PIB-PET

**Fig 4:**
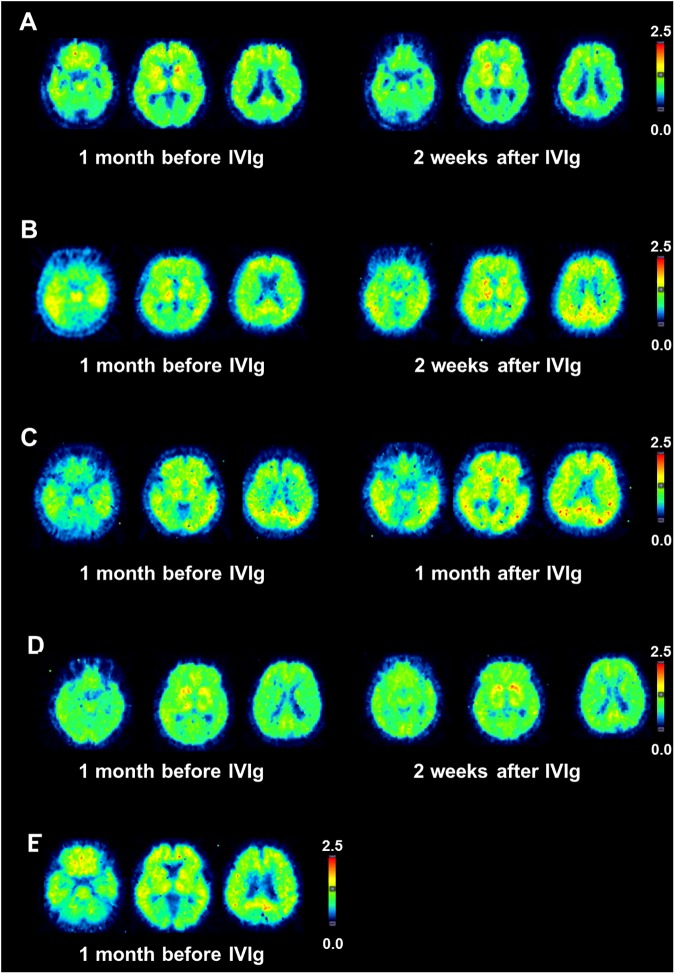
Transaxial PIB-PET scans before and after the IVIg treatment. DVR maps in each patient are shown. A, B, C, D, and E represent case 1, 2, 3, 4, and 5, respectively. Rate for PIB retention after the treatment was –6.7% in case 1,+10% in case 2, -5.6% in case 3 and +2.2% in case 4.

Aβ deposition measured by PIB-PET DVR map in each patient is shown in Fig 4. In all cases, PIB retention is observed in the frontal, posterior cingulate, striatum, parietal, and lateral temporal areas, as described previously [22]. There was no apparent change in distribution pattern before and after the treatment. The MCDVR was 1.38 (before) / 1.31 (after) in case 1, 1.53 (before) and 1.69 (after) in case 2, 1.79 (before) / 1.69 (after) in case 3, 1.36 (before) /1.39 (after) in case 4, and 1.55 (before) in case 5. Rate of changes for PIB retention in all participants after the treatment (calculated by the formula of MCDVR (after)–MCDVR (before)/ MCDVR (before)) were within the measurement error range in our institute (±10%). Therefore, we interpreted that no significant change in PIB retention was observed in any patient after the treatment compared with baseline.

### CSF and plasma biomarkers

**Fig 5:**
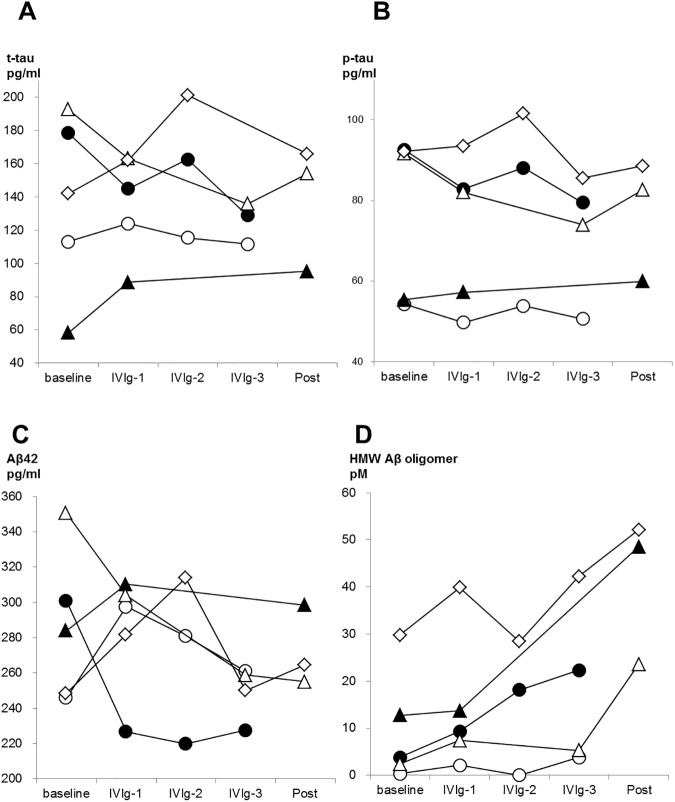
Changes of CSF biomarkers over the period of treatment. Case 1, 2, 3 (good responders), 4 (a poor responder) and 5 (case with discontinued treatment) are represented by white circles, white triangles, white squares, black circles, and black triangles, respectively. A: t-tau B: p-tau C: Aβ42 D: HMW Aβ oligomers.

**Fig 6:**
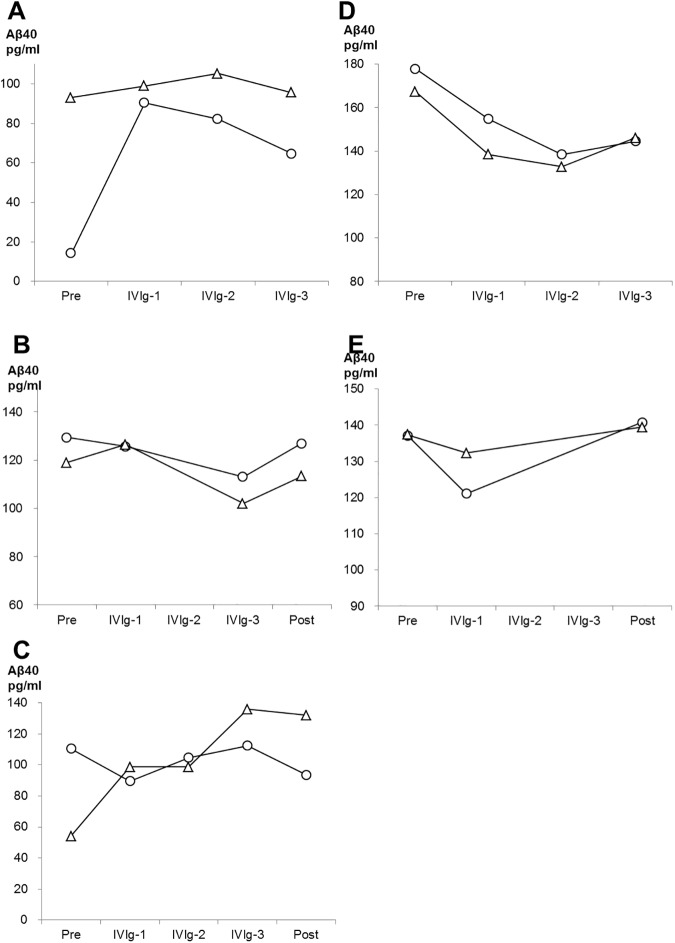
Changes of Aβ40 in peripheral-plasma and jugular-plasma over the period of treatment in the participants. Levels of Aβ40 in peripheral-plasma and jugular-plasma in each participant are represented by white circles and white triangles, respectively. The data from cases 1, 2, 3 (good responders), 4 (a poor responder) and 5 (case with discontinued treatment) are shown in A, B, C, D, and E, respectively.

**Fig 7:**
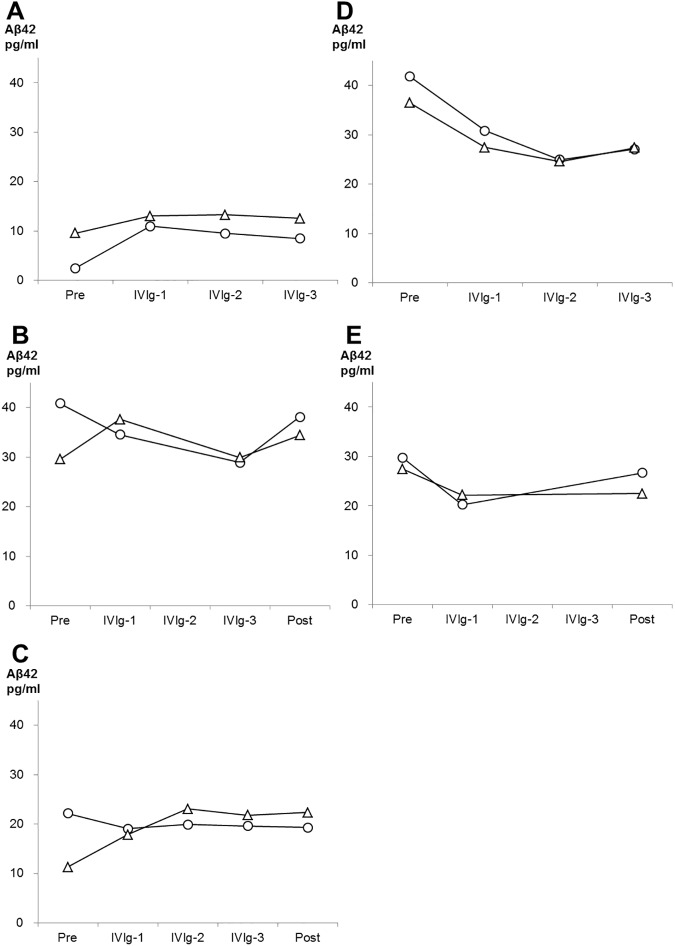
Changes of Aβ42 in peripheral-plasma and jugular-plasma over the period of treatment in the participants. Levels of Aβ42 in peripheral-plasma and jugular-plasma in each participant are represented by white circles and white triangles, respectively. The data from cases 1, 2, 3 (good responders), 4 (a poor responder) and 5 (case with discontinued treatment) are shown in A, B, C, D, and E, respectively.

Levels of t-tau, p-tau, Aβ_42,_ and HMW Aβ oligomers in CSF are shown in Fig 5. T-tau and Aβ_42_ did not show any significant difference compared to baseline, at every cycle of IVIg, and at post treatment. On p-tau, four of five patients showed lower p-tau levels after the treatment than baseline, although the difference did not reach statistical significance (P = 0.315). (It is possible that difference did not reach significance due to our small sample size, because statistical power in this analysis was insufficient (0.106).) The level of HMW Aβ oligomers was significantly increased after the treatment (P = 0.043). Note, in case 1 and 4 who refused CSF examination at post treatment, the values at the third cycle of treatment were used for analysis above described. Regarding peripheral and jugular-plasma, the IVIg treatment altered neither Aβ_40_ nor Aβ_42_ levels (S1 Fig). As shown in the Figs 6 and 7, Aβ_40_ or Aβ_42_ levels in jugular-plasma were not always higher than those in peripheral-plasma at baseline, although the jugular vein is a major drainage route from the brain. Interestingly, in three good responders, Aβ_40_ and Aβ_42_ levels were elevated in jugular-plasma after the treatment. Furthermore, these increased Aβ_40_ and Aβ_42_ levels in jugular-plasma were maintained during the whole treatment period including post IVIg in cases 1 and 3. Case 2 showed a transient increase of these markers in the first cycle of IVIg. This elevation was more evident in Aβ_42_ than that in Aβ_40_. On the other hand, the poor responder (case 4) or the participant with suspension of the treatment (case 5) did not show any increase of Aβ levels in jugular-plasma. In contrast, the levels of Aβ_40_ and Aβ_42_ in peripheral-plasma over the treatment did not change in relation to reactivity to IVIg. (The raw data of the biomarkers are summarized in Table B,C,D,E,F,and G in S4 File)

## Discussion

Our first major finding is that the levels of HMW Aβ oligomers as measured by the BAN50 SAS-ELISA were significantly increased after treatment in all participants. Regarding monomeric Aβ species, we found that IVIg therapy altered the levels of neither Aβ_40_ nor Aβ_42_ in CSF, which is in accord with previous results [23]. Aβ oligomers in CSF have not been adopted as a biomarker in previous studies of IVIg or other immunotherapies for AD due to the poor reliability of measurement. The BAN50 SAS-ELISA that we have developed is one of the few systems available that can reliably and reproducibly detect HMW Aβ oligomers in CSF [24]. The result was reproduced using the same set of antibody in one following study [25]. Our data suggest that HMW Aβ oligomers could be a better CSF biomarker to reflect the anti-amyloid effects of IVIg in the brain than Aβ_40_ or Aβ_42_. The increase in HMW Aβ oligomers in CSF on IVIg treatment could be due to the disaggregation of Aβ fibrils by the anti-Aβ antibodies contained in IVIg [26]. Moreover, we should note the other possibility that an increased signal in the BAN50-SAS ELISA could also be derived not from genuine ‘pathological’ Aβ oligomers (consisting only of Aβ), but also from complexes composed of multiple copies of Aβ in association with other molecules which, in theory at least, would also be detected by this ELISA [16, 24]. IVIg treatment is reported to increase the levels of anti-Aβ antibodies in CSF [11]. Therefore, pseudo (or non-pathological) oligomer complexes consisting of multiple copies of Aβ cross-linked by such antibodies could possibly be detected in this study.

Three of the five patients had preserved cognitive function more than one year after the IVIg treatment and all three of them showed sustained or transiently increased Aβ_40_ and Aβ_42_ levels in jugular-plasma after the treatment. IVIg has been reported not to alter the levels of Aβ_40_ or Aβ_42_ in peripheral-plasma [23], which is in agreement with our result that Aβ species in peripheral-plasma did not change in response to treatment. These results suggest that the levels of Aβ in peripheral-plasma are not a good measure for estimation of the efflux of Aβ from the brain in immunotherapy of AD. The levels of Aβ species in jugular-plasma after passive immunization therapy for AD have not been measured previously. Our results imply that Aβ species in jugular-plasma will provide a more direct and precise biomarker for estimation of Aβ clearance from the brain in the study of IVIg or other passive immunotherapies than those in peripheral-plasma. Moreover, our data provide some supportive evidence that IVIg may be more beneficial in AD in cases with efficient clearance of Aβ from the brain to the jugular vein than in those with poor clearance of Aβ.

In this study, the levels of Aβ in jugular-plasma were not always higher than those in peripheral-plasma, even though the internal jugular veins drain most of the outflow from the brain and so would be expected to be a major source of brain-derived Aβ. We suppose that this unexpected result is probably explained by the following two factors. The first is that Aβ is also produced from peripheral sources outside of the brain. Plasma Aβ is derived from not only the central nervous system, but also from the liver or from arteries with atherosclerosis [27]. Such systemically derived plasma Aβ might play a confounding role in this study. The second factor is the possibility that drainage of Aβ from the brain could be mainly or partly via another route instead of the jugular vein. Recent reports show that there are drainage pathways of brain interstitial Aβ along the cerebral vessels into cervical lymph nodes, and finally superior vena cava via the thoracic duct [28, 29]. Such lymphatic drainage might explain the imbalance between Aβ levels in peripheral and jugular-plasma.

We also found that Aβ deposition as measured by PIB-PET was not altered by the IVIg treatment. It is still controversial whether passive immunization can improve PIB retention [8, 30]. Moreover, regarding IVIg therapy, Relkin et. al. reported no significant changes in PIB retention for 6 months in the brains of 12 patients with AD treated with IVIg [31]. Moreover, one report in a transgenic mouse model of AD showed that naturally occurring autoantibodies against Aβ preferentially bind to Aβ oligomers but fail to bind to monomers or fibrils and therefore, improved memory but did not readily clear senile plaques from the brains of transgenic mice [32]. The lack of reduction in PIB retention in our study is consistent with these previous results. These facts suggest that disaggregation of Aβ fibril by the IVIg therapy has a too small to reduce Aβ deposition in the brain in such a short period and, therefore, that PIB retention is not a good marker to reflect the anti-amyloid effects of IVIg.

## Conclusions

The present study has found that treatment with IVIg resulted in an increase in the levels of HMW Aβ oligomers, as measured by the BAN50 SAS-ELISA, in all participants; Aβ_40_ and Aβ_42_ levels in jugular-plasma showed a transient or sustained increase after treatment in three of five patients, which correlated with preserved cognitive function, whereas Aβ levels in peripheral-plasma did not correlate with any treatment response; and PIB retention was not changed after IVIg treatment. These results suggest that the levels of HMW Aβ oligomers in CSF give a better indication of the anti-amyloid effects of IVIg than more conventional measures of CSF Aβ; Aβ in jugular-plasma possibly provides a more direct and precise measure of clearance of Aβ from the brain in immunotherapy trials than peripheral-plasma; and PIB-PET appears to be unsuitable for estimation of the anti-amyloid effects of immunotherapy. Although the small sample size in our study limits these conclusions, these findings do have positive implications for development of a surrogate biomarker to measure the efficacy of the immunotherapy, including IVIg therapy, for future clinical studies of AD.

## Supporting information

S1 Fig Changes of plasma biomarkers over the period of treatment. Case 1, 2, 3 (good responders), 4 (a poor responder) and 5 (case with discontinued treatment) are represented by white circles, white triangles, white squares, black circles, and black triangles, respectively. n.s: not significant. A: Aβ_40_ in peripheral-plasma B: Aβ_40_ in jugular-plasma C: Aβ_42_ in peripheral-plasma D: Aβ_42_ in jugular-plasma.
(PPT)

S1 File TREND Statement Check list of this study. (PDF)

S2 File Research implementation plan written by Japanese. (PDF)

S3 File Research implementation plan translated to English. (DOC)

S4 File Raw data of this study. Table A in S4 File. Summary of the clinical course after IVIg therapy in the 5 patients. Table B in S4 file. Changes for t-tau concentration in CSF (pg/ml). Table C in S4 File. Changes for p-tau concentration in CSF (pg/ml). Table D in S4 File. Changes for Aβ42 concentration in CSF (pg/ml). Table E in S4 File. Changes for HMW Aβ oligomers levels in CSF (pM). Table F in S4 File. (a). Changes for Aβ40 concentration in peripheral-plasma (pg/ml) (b). Changes for Aβ40 concentration in jugularl-plasma (pg/ml). Table G in S4 File. (a). Changes for Aβ42 concentration in peripheral-plasma (pg/ml) (b). Changes for Aβ42 concentration in jugularl-plasma (pg/ml).
(DOCX)
