# Supplementary figures and images for: Aβ levels in the jugular vein and high molecular weight Aβ oligomer levels in CSF can be used as biomarkers to indicate the anti-amyloid effect of IVIg for Alzheimer’s disease
Source: PLoS One. 2017 Apr 10;12(4):e0174630. doi: 10.1371/journal.pone.0174630 (PMC5386327; doi:10.1371/journal.pone.0174630)

## Slide 1
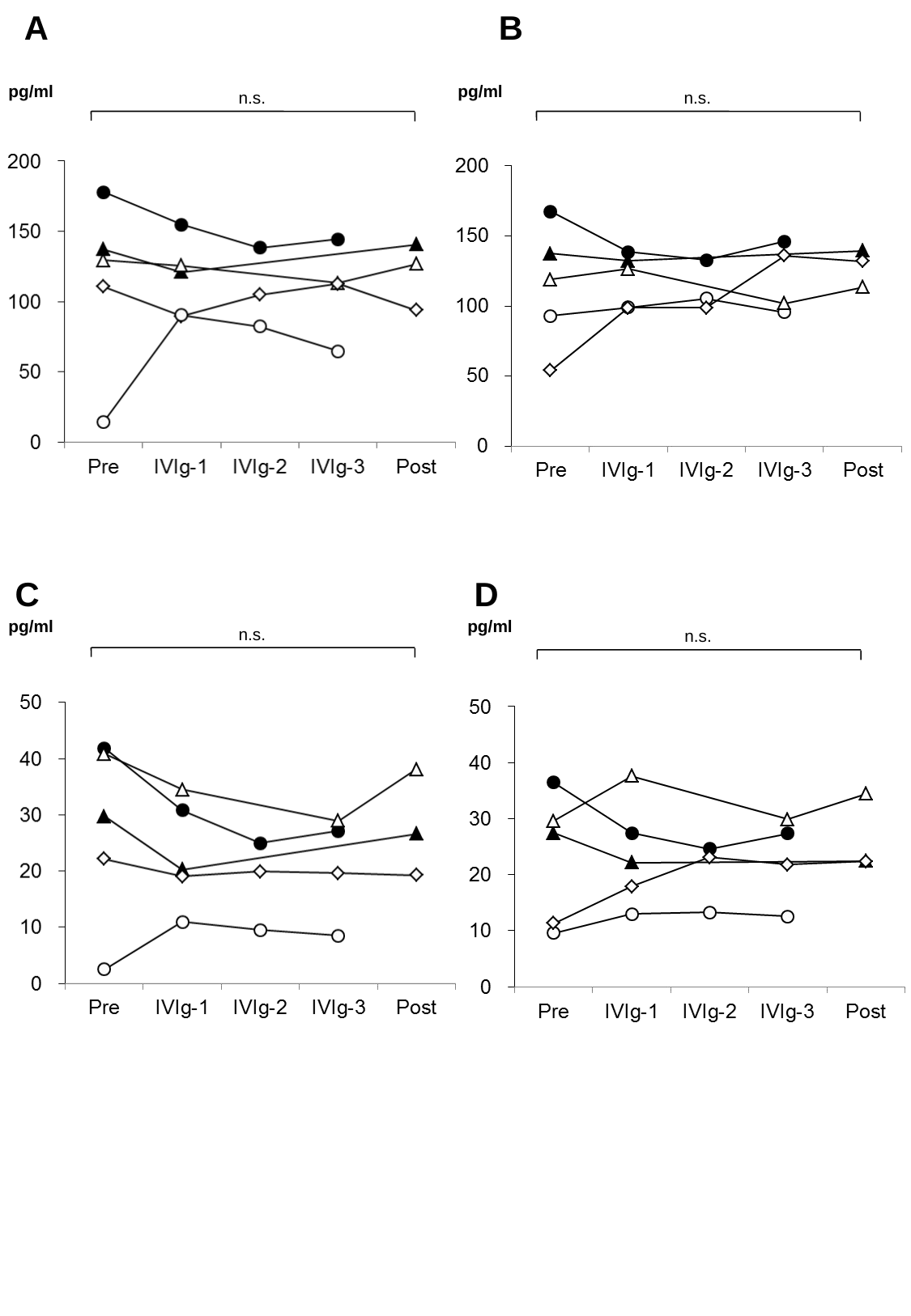

A
B
pg/ml
pg/ml
n.s.
n.s.
C
D
pg/ml
pg/ml
n.s.
n.s.

Supplement: S1 Fig — Case 1, 2, 3 (good responders), 4 (a poor responder) and 5 (case with discontinued treatment) are represented by white circles, white triangles, white squares, black circles, and black triangles, respectively. n.s: not significant. A: Aβ40 in peripheral-plasma B: Aβ40 in jugular-plasma C: Aβ42 in peripheral-plasma D: Aβ42 in jugular-plasma. (PPT) [file pone.0174630.s001.ppt]
