# Supplementary material for: Aβ levels in the jugular vein and high molecular weight Aβ oligomer levels in CSF can be used as biomarkers to indicate the anti-amyloid effect of IVIg for Alzheimer’s disease
Source: PLoS One. 2017 Apr 10;12(4):e0174630. doi: 10.1371/journal.pone.0174630 (PMC5386327; doi:10.1371/journal.pone.0174630)
Supplement: S2 File — (PDF) [file pone.0174630.s003.pdf]

実　施　計　画

1　課題名　アルツハイマー病関連蛋白質の脳内除去過程の検討

2　実施計画の意義，目的，方法（対象とする疾患，分析方法等．単一遺伝子疾患等の場合には研究の必要性，不利益を防止するための措置等の特記事項等）

アミロイドβ蛋白（Aβ）の沈着がアルツハイマー病病態の初期の段階に拘わっていることは既に明らかとなっている．Aβの沈着は脳内での産生と除去のそれぞれの過程での病態が考えられる．Aβの産生に関する報告は多くみられているが，除去過程に関する報告は少ない．除去の過程にはAβの脳内分解と脳からの運び出しが想定され，脳内分解については近年ネプリライシン等に関する報告がなされてきており研究が進められてきているが，脳からの運び出しの過程は現在のところほとんど調べられていない．しかし，松原らはアルツハイマー病モデルマウスで頸部リンパ節にAβの沈着がみられることを報告しており，Aβの脳からの運び出し過程についての臨床的な検討は重要と考えられる．また，アルツハイマー病治療は多角的に研究が進められているが未だ不十分な段階にある．その中でアルツハイマー病治療の一環としてワクチン療法をはじめとした免疫療法が開発されてきている．ガンマグロブリン点滴は種々の神経免疫疾患に臨床利用され免疫修飾療法としての効果をあげており，アルツハイマー病治療についても血中に投与された抗Aβ蛋白抗体によるAβの脳からの運び出し過程を増幅する可能性が考えられる．

本研究ではアルツハイマー病およびアルツハイマー病前段階と考えられるMCIと診断された患者に対して，髄液および血液のAβ40およびAβ42の総量およびオリゴマーの割合を測定する．Aβ総量は市販の抗Aβ蛋白抗体を組み合わせたELISAキットを使い，Aβオリゴマーは国立長寿医療研究所の松原らが開発した抗Aβオリゴマー抗体を組み合わせたELISAキットを使用する．併せて髄液および血液のアルツハイマー病関連蛋白の測定を行う．脳からの運び出しの過程を詳細に検討するために血液の採血は通常の肘静脈からの採血に加えて脳からの流出血液が集まる内頚静脈での採血を行う．内頚静脈採血は安全性に実施するためにポータブル超音波装置を用いて内頚静脈の走行を同定した上で右内頚静脈穿刺により採血する．また，Aβの沈着に対する治療的試みとして同意を頂いた患者に対してガンマグロブリン点滴による免疫修飾治療を行う．ガンマグロブリン点滴は原則として入院で行う．髄液および血液の採取はその前後で実施して，ガンマグロブリン療法前後での脳からのAβの運び出しについて検討する．

3　実施期間

承認日～平成22年3月31日

4　予測される結果及び危険性

アルツハイマー病におけるAβの脳からの運び出し過程はほとんど調べられておらず，臨床的な検討も現在なされていない．本研究で得られる所見はアルツハイマー病の病態解明・治療薬開発の糸口になる可能性がある．

髄液および血液の採取のため，腰椎穿刺，静脈穿刺を要する．腰椎穿刺後に頭痛，腰痛を一時的に発症したり，内頚静脈穿刺にて内頚動脈を誤って穿刺した場合に血腫をきたす可能性がある．また，ガンマグロブリン点滴ではショック，過敏症，血栓・塞栓症，急性腎不全，無菌性髄膜炎などが報告されている．

5　個人情報の保護の方法

研究結果や成果を学会や論文で発表する際は，個人が特定できない配慮を行ない，提供者のプライバシーを守る．

6　研究対象者，試料提供者を選ぶ方針

病歴，神経心理検査，MRI，脳血流SPECTでアルツハイマー病およびアルツハイマー病前段階と考えられるMCIと診断された患者を研究対象とする．未発症者は対象としない．

7　研究対象者の内容（年齢，性別，人数），試料等の内容（種類，量）

研究対象者は20名，年齢・性別は問わない．

8　共同研究機関の名称

国立長寿医療センター研究所アルツハイマー病研究部　松原　悦朗
弘前大学医学部脳神経統御部門　東海林幹夫

9　実施責任者等の氏名

神経病態制御学　近藤正樹

10　インフォームド・コンセントのための手続及び方法

本研究への協力の承諾および人権保護，プライバシー保護について，本人に対して本研究の内容を所定の「説明書」を用いて説明し，拒否による不利益は生じないことも十分説明したうえで，本人の自由意志により文書による承諾を得る．また研究開始後も同意の撤回が可能であることを説明する．

11　他の研究機関から試料等又は遺伝情報の提供を受ける場合のインフォームド・コンセントの内容

外部機関から試料または臨床情報の提供は受けない．

12　試料等又は遺伝情報を外部の機関に提供する場合や研究の一部を委託する場合の匿名化

外部機関へ試料または臨床情報の提供はしない．

13　試料等の保存方法及びその必要性(他の研究への利用の可能性と予測される研究内容を含む)

該当しない．

14　ヒト細胞・遺伝子・組織バンクに試料等を提供する場合には，バンク名，匿名化の方法

該当しない．

15　遺伝カウンセリングの必要性及びその体制

該当しない．

16　資金の調達方法

主に文部科学省の科学研究費や厚生労働省の研究補助金などを研究資金とする．提供者および家族に金銭的負担をかけることはない．

（注）　様式は自由とするが，別紙「実施計画書に記載すべき事項」に基づき，必要な事項　を漏れなく記載すること．
