# Supplementary material for: Aβ levels in the jugular vein and high molecular weight Aβ oligomer levels in CSF can be used as biomarkers to indicate the anti-amyloid effect of IVIg for Alzheimer’s disease
Source: PLoS One. 2017 Apr 10;12(4):e0174630. doi: 10.1371/journal.pone.0174630 (PMC5386327; doi:10.1371/journal.pone.0174630)
Supplement: S3 File — (DOC) [file pone.0174630.s004.doc]

**Research Implementation Plan**

**1 Title: Investigation of clearance of amyloid beta from the brain by IVIg**

**2 Significance, purpose, and methods of the research implementation plan (disease being studied, methods of analysis, etc. In the case of single-gene diseases, special notes on the necessity of the research and measures to prevent harm)**

Amyloid β protein (Aβ) deposits have been shown to be involved in the early stage of Alzheimer disease pathology. Aβ deposition is thought to be a pathological condition involving both production and elimination processes in the brain. There are many reports on Aβ production, but few on the elimination process. The elimination process is assumed to include the breakdown of Aβ in the brain and its transport from the brain, and while research on its breakdown in the brain has been progressing, with reports on neprilysin and other substances in recent years, to date there has been almost no investigation of the process of transport from the brain. However, Matsubara et al. reported that Aβ deposits are seen in the cervical lymph nodes in a murine model of Alzheimer’s disease, and it is believed that clinical investigation of the process of Aβ transport from the brain is important. Research on the treatment of Alzheimer’s disease is also progressing on many fronts, but it remains at an unsatisfactory level. In these circumstances, vaccine therapies and other immunotherapies are being developed as one part of Alzheimer’s disease treatment. Gamma globulin infusion is used clinically for various neuroimmune diseases, and it has shown some effectiveness as an immunomodulation therapy. In the treatment of Alzheimer’s disease as well, it is thought that anti-Aβ protein antibody administered systemically may amplify the process of transporting Aβ from the brain.

In this study, we will measure the total amount and percentage of oligomers of Aβ 40 and Aβ 42 in the spinal fluid and blood of patients diagnosed with Alzheimer’s disease and in those with mild cognitive impairment (MCI), which may be a prelude to Alzheimer’s disease. For the total amount of Aβ, we will use an ELISA kit combined with commercial anti-Aβ protein antibody, and for the Aβ oligomer we will use an ELISA kit combined with an anti-Aβ oligomer antibody developed by Matsubara and colleagues at the National Center for Geriatrics and Gerontology. We will also measure Alzheimer’s disease-related proteins in the spinal fluid and blood. Blood for a detailed investigation of the process of transport from the brain will be collected from the cubital vein, as usual, as well as from the internal jugular vein that gathers blood flowing out of the brain. To collect blood safely from the internal jugular vein, the course of the internal jugular vein will be identified using a portable ultrasound machine, after which the blood will be collected with puncture of the right internal jugular vein. Immunomodulation therapy with gamma globulin infusion as a therapeutic approach for Aβ deposition will also be conducted in patients who have given informed consent. The gamma globulin infusion shall, as a rule, be done with the patients hospitalized. Spinal fluid and blood will be collected before and after to investigate Aβ transport from the brain before and after gamma globulin therapy.

**3 Study duration**

Date of approval to March 31, 2010.

**4 Predicted results and risks**

The process of Aβ transport from the brain in Alzheimer’s disease has been the subject of almost no studies, and there have been no clinical investigations to date. The findings obtained in this study may provide clues for the elucidation of Alzheimer’s disease pathology and for the development of therapeutic agents.

Lumbar puncture and venipuncture are required for the collection of spinal fluid and blood. There is a possibility of transient headache or low back pain after lumbar puncture, and hematoma may occur in the event that the internal carotid artery is mistakenly punctured during the puncture of the internal jugular vein. With gamma globulin infusion, there have also been reports of shock, hypersensitivity, thrombosis/embolism, acute renal failure, aseptic meningitis, and other conditions.

**5 Method of protecting personal information**

When research results or outcomes are published at conferences or in journals, care will be taken so that individuals cannot be identified to protect the privacy of the subjects.

**6 Policy for selection of research subjects, specimen donors**

The subjects will be patients diagnosed with Alzheimer’s disease or MCI, which may be a prelude to Alzheimer’s disease, based on medical history, neuropsychological tests, MRI, and cerebral blood flow SPECT. Individuals without these conditions will not be recruited.

**7 Details on research subjects (age, sex, number) and specimens (type, amount)**

There will be 20 research subjects with no regard to age or sex.

**8 Names of collaborating research institutions**

Department of Alzheimer’s Disease Research, National Center for Geriatrics and Gerontology

Etsuro Matsubara

Department of Neurology, Hirosaki University School of Medicine

Mikio Shoji

**9 Name of implementation supervisor, etc.**

Department of Neurology

Masaki Kondo

**10 Procedures and methods for informed consent**

Individuals will be given an explanation, using a prescribed “explanation form” describing the details of this study, regarding consent to participate in this study, protection of their rights, and protection of privacy. After fully explaining that they will suffer no disadvantage from refusing, written, informed consent will be obtained based on the voluntary choice of the individuals. It will also be explained that they can withdraw their consent even after the study has begun.

**11 Details of informed consent in the case that specimens or genetic information is provided from other research institutions**

No specimens or clinical information provided by external institutions will be received.

**12 Anonymization in cases when specimens or genetic information is provided to external institutions or part of the study is outsourced**

No specimens or clinical information will be provided to external institutions.

**13 Method of preserving specimens, etc., and the necessity of doing so (including study content for which the possibility of use in other research is predicted)**

Not applicable.

**14 In cases when specimens etc. will be provided to a human cell, gene, or tissue bank, name of the bank and method of anonymization**

Not applicable.

**15 Need for genetic counseling and the system for it**

Not applicable.

**16 Method of procuring funds**

Main research funding will be from Grants-in-Aid for Scientific Research, Ministry of Education, Culture, Sports, Science and Technology and research grants from the Ministry of Health, Labour and Welfare. There will be no financial burden on donors or their families.

Note: Be sure to write down all necessary matters, without exception, based on the attachment “Matters to be disclosed in research implementation plan.” There is no prescribed format.
