# Supplementary material for: Aβ levels in the jugular vein and high molecular weight Aβ oligomer levels in CSF can be used as biomarkers to indicate the anti-amyloid effect of IVIg for Alzheimer’s disease
Source: PLoS One. 2017 Apr 10;12(4):e0174630. doi: 10.1371/journal.pone.0174630 (PMC5386327; doi:10.1371/journal.pone.0174630)
Supplement: S4 File — Table A in S4 File. Summary of the clinical course after IVIg therapy in the 5 patients. Table B in S4 file. Changes for t-tau concentration in CSF (pg/ml). Table C in S4 File. Changes for p-tau concentration in CSF (pg/ml). Table D in S4 File. Changes for Aβ42 concentration in CSF (pg/ml). Table E in S4 File. Changes for HMW Aβ oligomers levels in CSF (pM). Table F in S4 File. (a). Changes for Aβ40 concentration in peripheral-plasma (pg/ml) (b). Changes for Aβ40 concentration in jugularl-plasma (pg/ml). Table G in S4 File. (a). Changes for Aβ42 concentration in peripheral-plasma (pg/ml) (b). Changes for Aβ42 concentration in jugularl-plasma (pg/ml). (DOCX) [file pone.0174630.s005.docx]

**Supplementary Tables**

**Table A. Summary of the clinical course after IVIg therapy in the 5 patients**

Case 1

|  | baseline | 1st cycle of IVIg | 2nd cycle of IVIg | 3rd cycle of IVIg | Post IVIg |
| --- | --- | --- | --- | --- | --- |
| MMSE | 8 | 9 | 12 | 10 | 8 |
| ADAS-cog | 33.3 | 34.7 | 38.7 | 31 | 37.3 |
| CDR | 2 | 2 | 3 | 2 | 2 |
| FAST | 6c | 6c | 6c | 6c | 6c |

Case 2

|  | baseline | 1st cycle of IVIg | 2nd cycle of IVIg | 3rd cycle of IVIg | Post IVIg |
| --- | --- | --- | --- | --- | --- |
| MMSE | 17 | 22 | 25 | 26 | 22 |
| ADAS | 11.7 | 16 | 18.7 | 17.6 | 17.7 |
| CDR | 1 | 1 | 1 | 1 | 1 |
| FAST | 4 | 4 | 4 | 4 | 4 |

Case 3

|  | baseline | 1st cycle of IVIg | 2nd cycle of IVIg | 3rd cycle of IVIg | Post IVIg |
| --- | --- | --- | --- | --- | --- |
| MMSE | 23 | 26 | 26 | 18 | 25 |
| ADAS-cog | 18.3 | 10 | 10.7 | 10.3 | 8.4 |
| CDR | 0.5 | 0.5 | 0.5 | 0.5 | 0.5 |
| FAST | 3 | 3 | 3 | 3 | 3 |
| WMS-R LM I | 3 | 8 | 7 | 8 | 7 |
| WMS-R LM II | 0 | 0 | 0 | 0 | 0 |

Case 4

|  | baseline | 1st cycle of IVIg | 2nd cycle of IVIg | 3rd cycle of IVIg | Post IVIg |
| --- | --- | --- | --- | --- | --- |
| MMSE | 20 | 18 | 20 | 22 | 19 |
| ADAS-cog | 11.7 | 19 | 14.3 | 10.7 | 19.7 |
| CDR | 1 | 1 | 1 | 0.5 | 1 |
| FAST | 4 | 4 | 4 | 4 | 5 |

Case 5

|  | baseline | 1st cycle of IVIg | 2nd cycle of IVIg | 3rd cycle of IVIg | Post IVIg |
| --- | --- | --- | --- | --- | --- |
| MMSE | 25 | 29 | - | - | 28 |
| ADAS-cog | 3.6 | 3 | - | - | 5 |
| CDR | 0.5 | 0.5 | - | - | 0.5 |
| FAST | 3 | 3 | - | - | 3 |
| WMS-R LM I | 8 | 13 | - | - | 19 |
| WMS-R LM II | 3 | 13 | - | - | 13 |

WMS-R LM I: Wechsler Memory Scale Revised (WMS-R) logical memory I

WMS-R LM II: Wechsler Memory Scale Revised (WMS-R) logical memory II

**Table B. Changes for t-tau concentration in CSF (pg/ml)**

| Patient  No. | baseline | 1st cycle of IVIg | 2nd cycle of IVIg | 3rd cycle of IVIg | Post IVIg |
| --- | --- | --- | --- | --- | --- |
| 1 | 112.9 | 123.4 | 115.3 | 111.0 |  |
| 2 | 192.6 | 162.4 |  | 134.9 | 153.9 |
| 3 | 141.7 | 162.0 | 201.0 |  | 165.7 |
| 4 | 178.0 | 144.8 | 162.5 | 128.7 |  |
| 5 | 74.1 | 88.6 |  |  | 95.1 |

**Table C. Changes for p-tau concentration in CSF (pg/ml)**

| Patient  No. | baseline | 1st cycle of IVIg | 2nd cycle of IVIg | 3rd cycle of IVIg | Post IVIg |
| --- | --- | --- | --- | --- | --- |
| 1 | 54.3 | 49.8 | 53.9 | 50.6 |  |
| 2 | 91.7 | 81.7 |  | 74.0 | 82.6 |
| 3 | 92.0 | 93.3 | 101.5 | 85.5 | 88.5 |
| 4 | 92.5 | 82.9 | 88.1 | 79.4 |  |
| 5 | 55.4 | 57.2 |  | 59.9 | 63.9 |

**Table D. Changes for Aβ_42_ concentration in CSF (pg/ml)**

| Patient  No. | baseline | 1st cycle of IVIg | 2nd cycle of IVIg | 3rd cycle of IVIg | Post IVIg |
| --- | --- | --- | --- | --- | --- |
| 1 | 246.1 | 298.1 | 281.0 | 261.4 |  |
| 2 | 350.6 | 303.8 |  | 258.9 | 255.1 |
| 3 | 249.2 | 282.2 | 313.9 | 250.0 | 265.0 |
| 4 | 301.3 | 226.7 | 220.0 | 227.7 |  |
| 5 | 283.9 | 310.4 |  |  | 299.1 |

**Table E. Changes for HMW Aβ oligomers levels in CSF (pM)**

| Patient  No. | baseline | 1st cycle of IVIg | 2nd cycle of IVIg | 3rd cycle of IVIg | Post IVIg |
| --- | --- | --- | --- | --- | --- |
| 1 | 0.30 | 2.10 | 0 | 3.77 |  |
| 2 | 2.36 | 7.41 |  | 5.29 | 23.59 |
| 3 | 29.79 | 39.86 | 28.42 | 42.25 | 52.14 |
| 4 | 3.79 | 9.32 | 18.13 | 22.30 |  |
| 5 | 12.74 | 13.62 |  |  | 48.52 |

**Table F.**

**(a) Changes for Aβ_40_ concentration in peripheral-plasma** **(pg/ml)**

| Patient  No. | baseline | 1st cycle of IVIg | 2nd cycle of IVIg | 3rd cycle of IVIg | Post IVIg |
| --- | --- | --- | --- | --- | --- |
| 1 | 14.3 | 90.4 | 82.3 | 64.7 |  |
| 2 | 129.5 | 125.7 |  | 113.1 | 126.9 |
| 3 | 110.5 | 89.5 | 104.7 | 112.5 | 93.7 |
| 4 | 177.9 | 154.8 | 138.4 | 144.5 |  |
| 5 | 137.1 | 121.1 |  |  | 140.7 |

**(b) Changes for Aβ_40_ concentration in jugularl-plasma (pg/ml)**

| Patient  No. | baseline | 1st cycle of IVIg | 2nd cycle of IVIg | 3rd cycle of IVIg | Post IVIg |
| --- | --- | --- | --- | --- | --- |
| 1 | 93.1 | 99.1 | 105.3 | 95.6 |  |
| 2 | 119.0 | 126.4 |  | 102.0 | 113.4 |
| 3 | 54.1 | 98.6 | 98.6 | 135.9 | 132.1 |
| 4 | 167.4 | 138.5 | 132.8 | 146.0 |  |
| 5 | 137.4 | 132.3 |  |  | 139.5 |

**Table G.**

**(a) Changes for Aβ_42_ concentration in peripheral-plasma (pg/ml)**

| Patient  No. | baseline | 1st cycle of IVIg | 2nd cycle of IVIg | 3rd cycle of IVIg | Post IVIg |
| --- | --- | --- | --- | --- | --- |
| 1 | 2.47 | 10.96 | 9.51 | 8.49 |  |
| 2 | 40.87 | 34.53 |  | 28.94 | 38.13 |
| 3 | 22.19 | 19.08 | 19.93 | 19.61 | 19.30 |
| 4 | 41.86 | 30.84 | 24.96 | 27.11 |  |
| 5 | 29.77 | 20.25 |  |  | 26.66 |

**(b) Changes for Aβ_42_ concentration in jugularl-plasma (pg/ml)**

| Patient  No. | baseline | 1st cycle of IVIg | 2nd cycle of IVIg | 3rd cycle of IVIg | Post IVIg |
| --- | --- | --- | --- | --- | --- |
| 1 | 9.56 | 12.98 | 13.24 | 12.51 |  |
| 2 | 29.57 | 37.63 |  | 29.91 | 34.46 |
| 3 | 11.30 | 17.88 | 23.06 | 21.83 | 22.36 |
| 4 | 36.48 | 27.45 | 24.59 | 27.38 |  |
| 5 | 27.46 | 22.17 |  |  | 22.46 |
